# Community Use of Face Masks against the Spread of COVID-19

**DOI:** 10.3390/ijerph18063214

**Published:** 2021-03-19

**Authors:** Luciano Bubbico, Giuseppe Mastrangelo, Francesca Larese-Filon, Paolo Basso, Roberto Rigoli, Martina Maurelli, Salvatore Ferlito, Marco Capelli, Claudio Gisabella, Mohammad Javanbakht, Saverio Bellizzi, Luca Cegolon

**Affiliations:** 1 Sensori-Neural Disabilities Research Unit, INAPP, 00198 Rome, Italy; l.bubbico@inapp.org; 2 Department of Cardiac, Thoracic, Vascular Sciences & Public Health, Padua University, 35122 Padua, Italy; giuseppe.mastrangelo@unipd.it; 3 Occupational Medicine Unit, Department of Medicine, Surgery and Health Sciences, University of Trieste, 34127 Trieste, Italy; larese@units.it (F.L.-F.); paolo.basso@studenti.units.it (P.B.); 4 Microbiology Unit, Ca’ Foncello Hospital, Local Health Unit N.2 ‘Marca Trevigiana”, 31100 Treviso, Italy; roberto.rigoli@aulss2.veneto.it; 5 Section of Dermatology and Venereology, Department of Medicine, University of Verona, 37129 Verona, Italy; martina.maurelli@gmail.com; 6 Department of Surgical Medical Sciences and Advanced Technologies, School of Medicine, University of Catania, 95124 Catania, Italy; ferlito@unict.it; 7 Ear Nose and Throat (ENT) Department, CDI—Italian Diagnostic Centre, 20122 Milan, Italy; info@otorinocremona.it; 8 Public Health Department, Local Health Unit N.2 “Marca Trevigiana”, 31100 Treviso, Italy; claudio.gisabella@aulss2.veneto.it; 9 Nephrology and Urology Research Center, Baqiyatallah University of Medical Sciences, Tehran 1435916471, Iran; mhmjvbt81@gmail.com; 10 Partnership for Maternal, Newborn & Child Health, World Health Organization, 1200 Geneva, Switzerland; saverio.bellizzi@gmail.com

**Keywords:** SARS-CoV-2, COVID-19, transmissibility, nasal cavity, health protection, face masks

## Abstract

The role of face masks to prevent and control COVID-19 is critical, especially since asymptomatic or pre-symptomatic infected individuals can shed high loads of SARS-CoV-2 in the surrounding environment. In addition to being a two-way barrier to protect against virions droplets both in terms of “source control” (for the benefits of the community) and “physical protection” (for wearer), face masks also allow maintaining physiological temperatures and humidity of the nasal cavity and mouth, independently from the external environmental conditions. Beyond compromising the viral transmission speed, exposure to cold environments could have a detrimental effect on the host’s susceptibility to SARS-CoV-2. The innate human immune system becomes in fact weaker with cooler nose temperatures and thus more vulnerable to viral replication. Furthermore, there is evidence that warm, humid climates are associated with reduced spread of SARS-CoV-2, while cold dry conditions favor its stability and transmissibility. In the early stage of a viral infection, a physiological body temperature in the upper airways supports the innate immune system, endorsing the muco-ciliary clearance, inhibiting, or deactivating any first settlement of viruses. Face masks are therefore strongly recommended also outdoors, especially under cold weather conditions, not only as a physical barrier against the transmission of SARS-CoV-2, but also to prevent the rapid cooling of the nasal mucosa and the inhibition of the human innate defense of the upper airways.

## 1. Background

In January 2020, COVID-19, an epidemic sustained by a novel beta coronavirus, the severe acute respiratory syndrome coronavirus type 2 (SARS-CoV-2), was announced by the Chinese Government. The epidemic rapidly spread worldwide, to be subsequently declared a global pandemic by the World Health Organization (WHO) [1].

Beyond a safe and effective vaccine, containment measures against COVID-19 still rely also on enforcement of various health protection measures such as social distancing, frequent hand washing, use of personal protective equipment (PPE, i.e., gloves, face masks, among others) as well as on identification, treatment and isolation of active cases, quarantine of contacts, and various ad hoc additional limitations to business activities and freedom of movement of individuals.

Transmission of COVID-19 occurs mainly by inhalation of SARS-CoV-2 virions via droplets or aerosol viral particles. The nasal cavity is the main entry gateway for SARS-CoV-2, since nasal swabs from symptomatic or asymptomatic COVID-19 patients carry much higher viral concentrations than throat swabs [2]. In more than 90% of cases SARS-CoV-2 transmission occurs through the nasal mucosa or the naso-lacrimal duct draining into the nasal cavity. SARS-CoV-2 then multiplies in the epithelium of the upper respiratory tract before spreading down to pulmonary alveoli [3].

The expression of cell surface enzyme angiotensin-2 converting enzyme (ACE-2), which binds to SARS-CoV-2 spike protein promoting its internalization into target cells [4,5], was probed in the nasal epithelium among a cohort of 305 patients aged 4 to 60 years: adjusting for sex and asthma condition, ACE-2 expression was found to increase with age, offering a fertile ground for the high loads of SARS-CoV-2 virions present in the nasal cavity [6].

Since asymptomatic or pre-symptomatic infected individuals are estimated to account for more than 50% of those testing positives for COVID-19 and considering the former two categories can still shed high loads of SARS-CoV-2 in the surrounding environment [7,8,9,10], face masks provide primarily a “source control”, containing the expulsion of salivary mucus droplets from infected subjects, for the benefit of the community. Nonetheless, face masks also confer “personal protection”, protecting the wearer from the inhalation of respiratory virions droplets [11,12]. The public health benefit of community use of face masks increases with the proportion of people wearing them consistently and correctly, being the combined effect of “source control” and “personal protection” [11,12]. Experimental and epidemiological evidence endorses the use of community masks to reduce the spread of SARS-CoV-2 [12].

At the beginning of the COVID-19 pandemic the acceptance of face masks was negatively influenced by the strange feeling of wearing them, a sensation inversely related with increasing number of wearers, regardless if they were surgical, filtering face pieces type 2 (FFP2) or non-medical (cloth) masks [13]. Psychological (fear of stigma; misconceptions on their efficacy) and social (others wearing/not wearing a mask; respecting recommendations) aspects played a relevant role to reject the use of face masks, at least in the general population.

While a pre-registered experiment (*n* = 925) indicated that a voluntary policy would likely lead to insufficient compliance with mask wearing, serial cross-sectional data (14 April to 26 May 2020) from nearly 7000 German participants demonstrated that implementing mandatory face masks increased their actual use; moreover, mask wearing correlated positively with other protective behaviors against the spread of COVID-19 [14].

Therefore, in October 2020, in response to a resurge of the COVID-19 pandemic following a relative deflation over the summer, some European countries introduced more stringent infection, prevention and control (IPC) coercive measures, mandating of wearing face masks also outdoors, a rule facing strong opposition by some sectors of the general population.

In view of the above we examined the literature on the evidence of the impact of community use of face masks against the spread of COVID-19, reviewing their physiological protecting mechanisms (in addition to their function as a two-way physical barrier against virions droplets), such as: maintenance of physiological temperatures and humidity of the upper airways; sustaining the muco-ciliary clearance; supporting the innate immune system of the human respiratory tract, which in the early stage of a viral infection can inhibit or deactivate any first settlement of pathogens.

## 2. Results and Discussion

### 2.1. Ambient Temperature and Nasal Microclimate

The heat of a gas depends on its temperature and relative humidity (RH). Under cold weather conditions the human body maintains a physiological temperature by reducing the blood flow to extremities and diverting it to vital organs. The human nose acts as an air conditioner, thermo-regulating, humidifying, and protecting the upper airways [15]. The nasal mucosa is highly vascularized and has a dominant role on breathed air conditioning. Since it is mainly composed of cartilage with limited insulating fat, the nose is particularly sensitive to ambient temperature [16]. By stimulating the sympathetic receptors, nasal stimulation with cold dry air induces reflex responses in the respiratory system such as broncho-constriction and increased pre-capillary resistance of the nasal micro-circulation, thereby decreasing the blood flow in the nasal mucosa [17,18]. A reduction of temperature and blood flow in the nasal cavity was observed at laser Doppler flowmeter following peripheral 5 min exposure of the feet of healthy subjects to cold water [18]. Depending on the respiratory cycle, while inhalation of cold air triggers a conditioning process diminishing the temperature of the nasal mucosa, exposure to warm air determines opposite effects directly in the mucosa [19]. With prolonged cold exposure the temperature drop of the nasal mucosa may overtake the heating capacity of the human body, thus paving the way to hypothermia [16].

Indeed, it is well known that covering the nose and mouth by a scarf under low outdoor temperatures warms the face, increases the temperature of the upper airways and moisture the inhaled air. Likewise, the use of face masks reduces the loss of heat and moisture during re-breathing [20,21].

In 93 healthy (hospital staff) subjects free from any active disease, oral temperature was significantly higher 30 min after wearing a mask (36.73 °C) than while they were not wearing it (36.56 °C; *p* = 0.002, paired *t* test) [22]. The heating of the nasal cavity and upper airways in individuals wearing a face mask is predominantly due to the effect of warm air exhaled from the lungs [23]. The microclimate inside a respirator is in fact influenced more by the exhaled breath rather than the inhaled air [24]. Face masks passively warm and humidify each breath, reducing the effect of external cold air on the airways. During each inspiration, the nasal airways partially extract heat and moisture from the face mask [25].

Pulmonary inflammation stimulates blood circulation in the lung airways walls, heating the air [7]. Asthmatic patients reportedly have significantly higher exhaled breath temperature while wearing a disposable respirator [24] and their induced sputum has higher concentration of exhaled nitric oxide and eosinophil percentage. Furthermore, since it reduces the exhaled breath temperature in adult asthmatic patients, exhaled breath temperature has become a marker of management of asthmatic symptoms [26,27,28].

**Figure 1:**
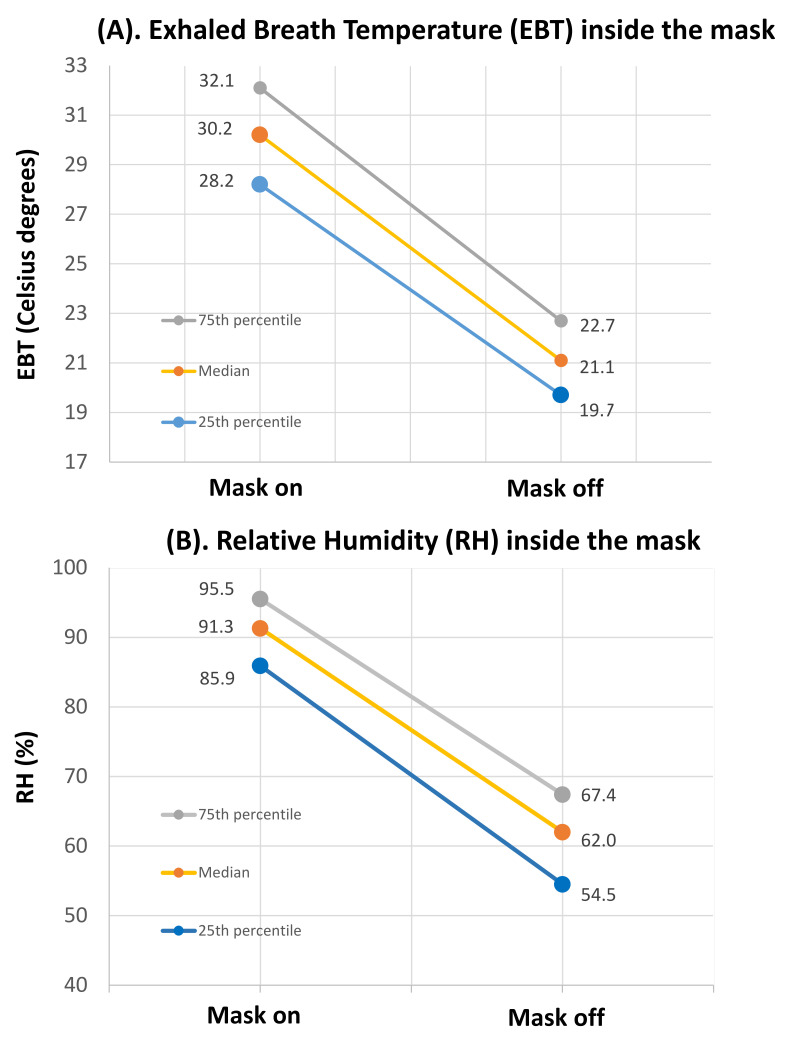
(**A**) From Cherrie et al. 2019 [29]. Exhaled Breath Temperature (EBT) inside the mask, measured by a sensor, when the disposable respirator (3M 9211) was worn (“Mask on”) or not (“Mask off”). Median EBT (in Celsius degrees) with interquartile range. (**B**) From Cherrie et al. 2019 [29]. Relative humidity (RH) inside the mask, measured by a sensor, when the disposable respirator (3M 9211) was worn (“Mask on”) or not (“Mask off”). Median RH (%) with interquartile range.

When disposable respirators are correctly fitted and donned, the microclimate—temperature and relative humidity (RH)—inside face masks reflects that of exhaled breathed air, rather than ambient conditions’ [29]. A recent study on nine volunteers from Central Scotland employed a novel small sensor (Hygrochron iButton^®^ DS1923, Maxim Integrated, San Jose, CA, US), located inside a disposable 3M 9211 respirator to data-log temperature and RH at regular intervals of 10 s, under supervised and unsupervised wearing trials [29]. Study subjects were allowed to wear the respirator wherever and whenever they wish for health protection during ordinary daily activities over the course of 5 days. As can be seen from Figure 1, under unsupervised wearing trial the temperature and RH of the exhaled air—measured when the mask was on—were substantially higher than the respective values when the mask was not worn [29].

**Figure 2:**
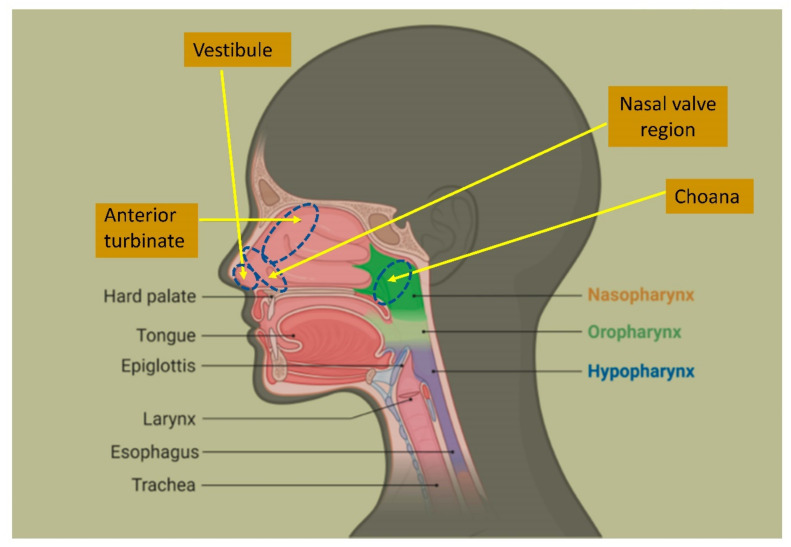
Anatomy of the nasal cavity.

**Figure 3:**
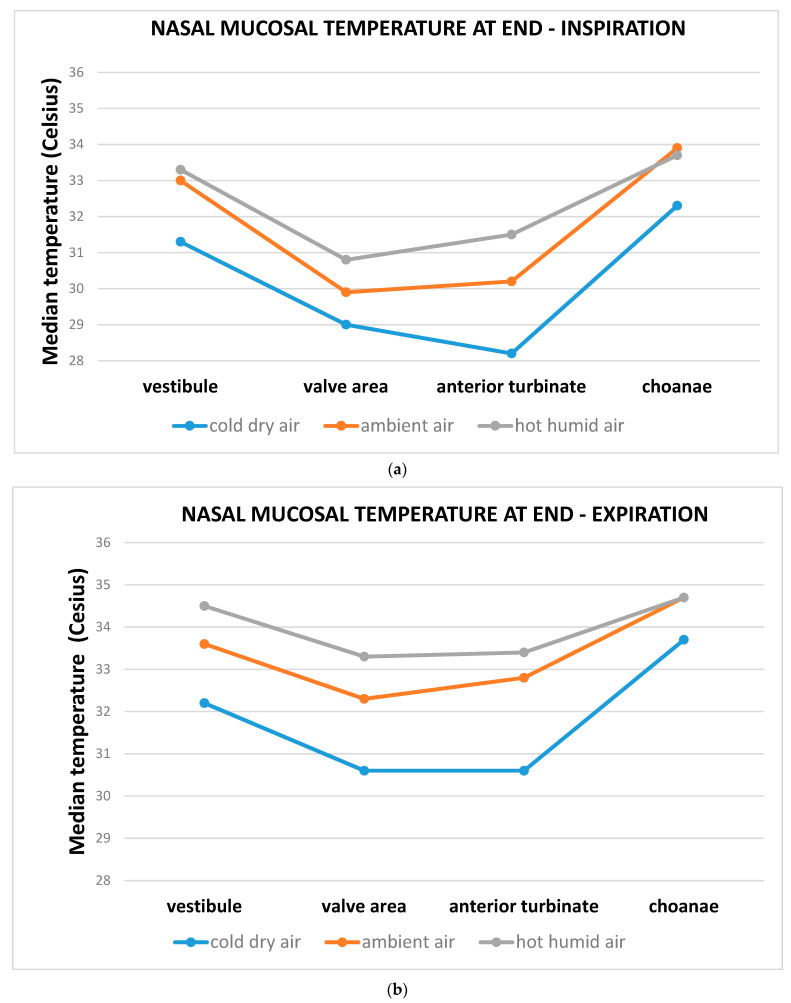
From Liener et al. 2020 [19]. Median mucosal temperatures (Celsius degrees) of four different anatomical nasal districts (vestibule, valve area, anterior turbinate, choanae) at end of inspiration (**a**) and expiration (**b**), after exposures to three different combinations of air temperature and relative humidity (RH), during three consecutive days. Continuous measurements made during a 2 min interval in which subjects inhaled ambient air (24 C; 35% RH)—**Day 1**: 10 min exposure to cold dry air (12 C; 13% RH); **Day 2**: 10 min exposure to ambient air (24 C; 35% RH); **Day 3**: 10 min exposure to hot humid air (40 C; 80% RH).

During three consecutive days, 15 healthy subjects (10 females vs. 5 males; average age 27 years; range 22–37) without history of nasal surgery or nasal trauma, were repeatedly exposed for 10 min to either: cold dry air (12 °C, 13% RH) on day 1; ambient air (24 °C; 35% RH) on day 2; hot humid air (40 °C; 80% RH) on day 3 [19]. Continuous temperature measurements were performed during a 2 min interval in which each subject could breathe calmly at ambient conditions (24 °C with 35% RH). The temperature of the nasal mucosa was measured by a miniaturized chrome-nickel-aluminum thermocouple placed in the right nasal cavity, from the anterior part of the nasal septum, from the nasal valve area, from the anterior turbinate and from the choanae. End-inspiration and end-expiration temperatures from a 1 min breathing cycle were analyzed. The climatic conditions of the inhaled air caused significant variations in the temperature of the nasal mucosal. End-expiration temperatures were higher than end-inspiration ones, hinting that the nasal mucosa receives heat and humidity from the exhaled air coming from the lungs. Moreover, a decreased temperature mucosal gradient was observed from the anterior to the posterior region of the nose, as a likely result of anatomical and histological differences between the two nasal districts. In the anterior third of the nose, the mucosa has in fact a variety of epithelial cell types and goblet cells are unevenly distributed. By contrast, the posterior portion of the nasal septum has higher density and a reduced number of seromucous glands. Furthermore, end-inspiration and end-expiration temperature readings varied in all nasal districts in the latter study, with end-expiration temperature being warmer than end-inspiration’s in all four districts, particularly in the anterior portion of the nose (Figure 2 and Figure 3) [19].

By preserving the humidity and temperature of the upper airways, facemasks therefore substantially contribute to protect the respiratory mechanisms of thermo-regulation [23]. Optimal nasal air conditions are also necessary for the functional exchange of gases in the lungs, avoiding the alveolar drying that could result by a sudden cooling of the airways.

### 2.2. The Impact of Cold Temperature on the Innate Human Defense System of Upper Airways

The nasal muco-ciliary clearance is the first mucosal defense layer of the upper airways, responsible for removing infectious pathogens, particulates and gaseous material [30]. In healthy subjects, the nasal muco-ciliary clearance and the frequency of ciliary beats depends on the local microclimate, which in turns is influenced by ambient temperature, RH, or atmospheric pollution [31]. The clearance function of the muco-ciliary activity can be disturbed by lower temperatures and increased RH, by viral infections and by exposure to atmospheric pollutants such as sulfur dioxide, nitrogen dioxide, aldehydes, and tobacco smoke [31]. Interfering or blocking the mucus drainage stimulates the proliferation of infectious agents in the nasal cavity and their penetration into the respiratory mucosa [32]. The nose and upper airways are an easy access to the human body for respiratory micro-organisms, which can also exit from these cavities to infect further susceptible individuals [20,21].

Rhinovirus (RV), the most frequent causative agent of common cold as well as one of the most important causes of asthma exacerbations, replicates more easily at cooler mucosal temperatures. The underlying mechanism depends also on a temperature-dependent host antiviral response (RIG-I–like receptor–dependent interferon secretion), which was assessed by incubating mouse primary airways cells infected by RV at lower and higher temperatures [33]. Even brief exposure to cold can increase the levels of norepinephrine and cortisol, determining lymphocytosis, suppressing lympho-proliferative responses, and decreasing levels of Th1 cytokines and salivary IgA. Whether these changes lead to increased susceptibility to infection, as suggested by some epidemiological reports, remains to be ascertained [34]. However, in workplaces as slaughterhouses or food processing plants, working in cold environmental conditions weakens the human immune response against SARS-CoV-2, further contributing to COVID-19 outbreaks [35].

#### Cold Temperature and Respiratory Viruses

The virulence of a microorganism depends on the temperature sensitivity of its replication and therefore ultimately on the body temperature of the host [36]. Sensitivity to temperature can interfere/prevent (restrictive temperature) or facilitate (permissive temperature) the replication of a virus. The first cell cultures of an unknown virus, later identified as RV, were initially unsuccessful at 36 °C and it was only when the environmental temperature was reduced to 32 °C that viruses replicated efficiently [37]. Experimental studies in guinea pigs demonstrated that influenza virus transmission is strongly modulated by temperature and humidity [38]. Exposure of Mx1 congenic mice to dry air impaired the host (e.g., muco-ciliary clearance) and innate antiviral (diminished expression of IFN stimulated genes) defense against influenza infection, reduced tissue repair and inflicted caspase-dependent disease pathology [39]. RH is more likely than absolute humidity to modulate virus survival and transmission according to a mechanism based on droplet evaporation with an impact on droplet physics and chemistry [40].

Epidemic waves caused by SARS-CoV-2 and its newly emerging variants burst up during winter months [41]. As the other respiratory viruses, SARS-CoV-2 has a tendency to colonize the upper airways, particularly the nasal cavity, where the temperature is lower than the core human body’s [20,21,42]. Overall, a temperature increase of the nasal cavity and the upper airways interferes with the replication and spread of SARS-CoV-2 [20]. In nasal mucus and sputum SARS-CoV-2 was more stable at low temperatures and low RH, whereas warmer temperatures and higher RH shortened its half-life [43]. Recent evidence confirmed that SARS-CoV-2 is sensitive to ambient temperature [44] as well as to RH and irradiation [45]. Considering the existing scientific evidence, warm and wet climates seem to reduce the spread of COVID-19. However, these variables alone could not explain most of the variability in disease transmission. Therefore, countries most affected by the COVID-19 should focus on health protection policies, even with climates less favorable to the virus [9,41,46].

Following SARS-CoV-2 infection of upper airways, an initial hyper-pyrexia is a host reaction against the viral spread. However, this immunological reaction may not be sufficient to heat the external ports of entry of the human body. Therefore, the protective function of face masks and scarfs against pathogens threats is integrated by a “therapeutic” one, linked to their role in heating the upper airways, generating an unfavorable microclimate for the settlement and spread of SARS-CoV-2 [20,21,23]. Facemasks and cloth face coverings should therefore be considered plain non-pharmacological devices to curb the spread of COVID-19 [20,21].

### 2.3. SARS-CoV-2, Allergy and Face Masks

Inhalation of cold air stimulates the inflammation of bronchial airways, thereby worsening conditions as asthma and chronic obstructive respiratory diseases (COPD) more in general. Inhalation of cold air is a risk factor for respiratory diseases and has negative effects on patients’ lungs, particularly among asthmatics [47]. Face masks already proved beneficial to decrease the risk of asthma stimulated by low ambient temperatures [48].

Surgical face masks filter particles larger than 3 mm, such as pollen (10–100 mm), fungal spores (2–50 mm), and house-dust mites feces (10–40 mm), which play a significant role in triggering IgE-mediated immunologic responses with typical allergic rhinitis symptoms [49]. Rhinitis symptoms were reported to be significantly reduced during the COVID-19 pandemic among nurses with seasonal allergic rhinitis wearing face masks. The contribution of face masks was confirmed by the lack of ophthalmic symptom improvement in these nurses, since their eye’s conjunctiva remained exposed to provoking allergens. Mask use based on personal allergen profiles can be considered a preventive measure to minimize exposure of the respiratory system to key allergens in high-risk environments [50]. Viral infections and atopic diseases are closely related and contribute to each other. Allergic diseases might predispose to viral infections or to a deferred viral clearance due to delayed and deficient production of the innate type I and type III interferons and/or deficient epithelial barrier function [50]. Viral infections, on the other hand, can induce several immunological mechanisms involved in allergic inflammation capable of promoting the initiation or exacerbation of atopic diseases such as atopic asthma [51]. However, further scientific evidence is needed to answer the research question as to whether allergic diseases per se or their treatment might predispose the respective patients to COVID-19 development and worse disease course.

#### 2.3.1. SARS-CoV-2, Air Pollution, Blood Pressure and Face Masks

FFP2/FFP3 respirators protect against particulate matter (PM) pollution, which in turn strengthens the transmissibility of SARS-CoV-2 [52]. PM in fact induces inflammation of respiratory cells, thus increasing the patient’s susceptibility to SARS-CoV-2 and the severity of COVID-19 symptoms [52]. Randomized cross-over trials conducted in Chinese cities of Beijing and Shanghai have also reported reduction of blood pressure and increased heart rate variability parameters from wearing a disposable respirator for 48 h [53] or for a few hours while walking [54,55], as a likely result of reduced exposure to ambient particulate concentrations. However, inhalation of cold, dry air can also increase blood pressure, enhancing the risk of myocardial infarction, particularly among individuals affected by pre-existing hypertensive cardiovascular disorders [56]. Therefore, considering the influence of cold air on systolic blood pressure, the use of facemasks against COVID-19 may also mitigate the effect of hypertension on the risk of myocardial ischemia, particularly in people with pre-existing hypertensive cardiovascular disease [56].

##### Filtering Efficacy of Face Masks

Face masks remove chemical and biological agents from the inhaled airflow, with an efficacy depending on the efficiency of its filter and the consistency of the “edge seal leakage”, the seal between the respirator and the skin face of the wearer [24,30].

The evidence of protection efficacy varies by type of face masks, being low to moderate for medical face masks and very low for non-medical ones made of cloth or textile materials [57].

One randomized control trial (RCT) comparing community transmission of COVID-19 between 3000 subjects wearing face masks against 3000 controls provided a non-significant 18% reduction of disease incidence in the intervention group [58]. Further case control or cross-sectional studies estimated stronger significant protection attributable to face masks against the transmission of COVID-19 in the community (OR from 0.16 to 0.30) and in health care settings. However, the evidence of the latter observational studies was limited by several bias including study design, different study settings, selection bias, recall bias or no distinction between cloth masks, surgical masks, and respirators [57]. The Institute for Health and Metrics Evaluation estimated a reduction by at least one third of COVID-19 transmission thanks to the use of disposable face masks [59].

Filtering face piece (FFP) are featured by higher filtration efficacy than medical masks. The capacity to filter 0.3 μm particles is 94% for FFP2 and 99% for FFP3 [57,60]. FFP are now recommended for their higher filtering capacity against more contagious variants of SARS-CoV-2, with a protection efficacy against community transmission of COVID-19 estimated to range from small to moderate [57].

Although respirators provide the highest level of protection thanks to their filtering material composition, efficacy variation was reportedly greatest for FFP2 than for surgical and homemade cloth masks, implying that skin fit is a key factor [29]. Nevertheless, any type of mask is likely to decrease viral exposure and infection risk on a population level, despite imperfect fit and adherence [61].

#### 2.3.2. Adverse Effects and Acceptability of Face Masks

Adverse effects have been reported in health care workers (HCW) wearing surgical masks indoor for more than 4 h a day [62,63,64]. These adverse effects include skin rashes, allergic contact dermatitis by rubber additives (carbamates, thiurams, mercapto-benziathiazoles [63]), contact urticaria by latex, seborrheic dermatitis and acne by increased sebum secretion induced by high skin temperature associated with face mask use [65,66].

In a recent online survey on 1156 HCW from Poland, 31.4% reported itch associated with wearing face masks. Prevalence of itch was more likely in HCW than controls (students) and increased significantly with face mask use (especially >4 h per day) and among females [63]. Risk factors for the development of itch related to face mask include skin sensitiveness, atopic predisposition, facial dermatoses (e.g., acne), atopic dermatitis or seborrhoeic dermatitis. Subjects affected by the latter conditions reportedly scratch their face without removing the mask or after removing it, which reduces protection of the mask [67].

Change of skin temperature, hydration, sebum secretion and excessive skin pressure caused by tight-fitting disposable respirators can determine cutaneous lesions as indentations of the face, skin tears, post-inflammatory hyperpigmentation, ulceration, crusting, erythema, and infection [68]. The risk of adverse skin reaction is more likely with surgical than cloth masks—OR = 1.54 (95%CI: 1.16–2.06)—[63].

A further critical aspect to be considered is the potential onset of hypercapnia associated with face mask use in patients affected by COPD [69].

The comfort of a disposable respirator is predominantly influenced by the microclimate inside it. In a cool environment, a mask temperature greater than or equal to 27 °C was reportedly fully comfortable among 6 study subjects cycling on an ergometer for 15 min [66]. Mask temperatures < 33 °C were 100% acceptable among 6 sedentary subjects, whereas higher respirator temperatures or higher RH reduced the acceptance rate [70]. Although physical exercise may lower the acceptability rate of face masks conditions, in a recent randomized cross-over trial wearing a face mask during cycle ergometry test to exhaustion had no impact on blood or muscle oxygenation and physical performance among 14 young healthy participants [71].

Work in heat (40–50 °C bulb temperature) caused discomfort when inspired air was slightly higher than body temperature (37 °C) [72]. By contrast, men at rest breathing warm moist air in a cool environment did not experience discomfort until the temperature of the inhaled air hit 54.5–63 °C [73].

Despite potential adverse effects due to a prolonged use, some simple devices could increase compliance with face masks and their acceptability: frequent breaks; hydration [74]; prophylactic dressing [75]; skin care and potentially newly designed comfortable respirators [76]. Since removal can immediately lower temperature and RH inside facemasks, duffing intervals should be considered, without wasting the protecting function of face masks [20].

## 3. Conclusions

The COVID-19 pandemic has imposed many social restrictions and behavioral coercive measures.

Consistent and correct use of face masks—also outdoors—remains one of the few critical non-pharmaceutical measures to protect the wearer from SARS-CoV-2, containing also the spread of COVID-19 in the community [20,21]. Face masks might play a further synergic role as a safeguarding public and occupational health measure, especially under cold weather conditions, preventing the rapid cooling of the nasal mucosa and the inhibition of the human innate defense of the upper airways.

Although the US Center for Disease Control and Prevention (CDC) recommends community use of face masks both indoors and outdoors to protect against COVID-19 [12], an intolerance due to itch and accrued facial heat caused by face masks may push toward their improper application or even reluctance, with consequent reduced protection for the wearer and the community [23,77]. Since the acceptability of disposable respirators varies importantly by both warmth and moisture of mask air [23,78], strategies to mitigate the microclimate associated with face mask (including duffing intervals) should be considered to increase their tolerance, with greater protection for the wearer and the community.

## Data Availability

No new data were created or analyzed in this study. Data sharing is not applicable to this article.
